# Tumorigenic Properties of Iron Regulatory Protein 2 (IRP2) Mediated by Its Specific 73-Amino Acids Insert

**DOI:** 10.1371/journal.pone.0010163

**Published:** 2010-04-13

**Authors:** Carmen Maffettone, Guohua Chen, Ignat Drozdov, Christos Ouzounis, Kostas Pantopoulos

**Affiliations:** 1 Lady Davis Institute for Medical Research, Sir Mortimer B. Davis Jewish General Hospital, and Department of Medicine, McGill University, Montreal, Quebec, Canada; 2 Cardiovascular Division, King's College London British Heart Foundation (BHF) Centre of Excellence, London, England, United Kingdom; 3 Centre for Bioinformatics, School of Physical Sciences & Engineering, King's College London, London, England, United Kingdom; Texas A&M University, United States of America

## Abstract

Iron regulatory proteins, IRP1 and IRP2, bind to mRNAs harboring iron responsive elements and control their expression. IRPs may also perform additional functions. Thus, IRP1 exhibited apparent tumor suppressor properties in a tumor xenograft model. Here we examined the effects of IRP2 in a similar setting. Human H1299 lung cancer cells or clones engineered for tetracycline-inducible expression of wild type IRP2, or the deletion mutant IRP2_Δ73_ (lacking a specific insert of 73 amino acids), were injected subcutaneously into nude mice. The induction of IRP2 profoundly stimulated the growth of tumor xenografts, and this response was blunted by addition of tetracycline in the drinking water of the animals, to turnoff the IRP2 transgene. Interestingly, IRP2_Δ73_ failed to promote tumor growth above control levels. As expected, xenografts expressing the IRP2 transgene exhibited high levels of transferrin receptor 1 (TfR1); however, the expression of other known IRP targets was not affected. Moreover, these xenografts manifested increased c-MYC levels and ERK1/2 phosphorylation. A microarray analysis identified distinct gene expression patterns between control and tumors containing IRP2 or IRP1 transgenes. By contrast, gene expression profiles of control and IRP2_Δ73_-related tumors were more similar, consistently with their growth phenotype. Collectively, these data demonstrate an apparent pro-oncogenic activity of IRP2 that depends on its specific 73 amino acids insert, and provide further evidence for a link between IRPs and cancer biology.

## Introduction

IRP1 and IRP2 are homologous cytoplasmic proteins that post-transcriptionally regulate cellular iron metabolism [1], [2], [3]. In iron-starved cells, both IRPs are activated for binding to iron responsive elements (IREs) within the untranslated regions of several mRNAs and thereby modulate their stability or translation. Among other targets, IRPs control the expression of transferrin receptor 1 (TfR1) and ferritin, which are key proteins of cellular iron uptake and storage, respectively. The binding of IRPs stabilizes TfR1 mRNA and inhibits translation of H- and L-ferritin mRNAs, promoting homeostatic adaptation to iron deficiency. Likewise, IRPs control the stability or translation of IRE-containing mRNA isoforms of the iron transporters DMT1 [4] and ferroportin [5].

IRPs are ubiquitously expressed in mammalian tissues and share at least some degree of functional redundancy. This is evident from the early embryonic lethality at the blastocyst stage, associated with the ablation of both IRP1 and IRP2 in mice [6]. Moreover, conditional disruption of both IRP1 and IRP2 in the mouse intestine elicits severe fatal pathology in this organ [7]. On the other hand, single IRP1-/- or IRP2-/- knockout mice are viable [8], [9], [10], [11]. Nevertheless, it appears that IRP2 has a more dominant role in vivo, considering that IRP1-/- mice are healthy [8], while IRP2-/- counterparts develop microcytic anemia [9], [11] and neurodegeneration [10]. In agreement with these findings, silencing experiments in HeLa cells identified IRP2 as the major regulator of TfR1 and ferritin mRNA expression, without, however, excluding a contribution of IRP1 [12].

In iron-replete cells, IRP1 assembles a cubane [4Fe-4S] cluster and acquires enzymatic function as cytosolic aconitase, at the expense of its RNA-binding activity [1], [2], while IRP2 undergoes ubiquitination by the E3 ligase FBXL5 and degradation by the proteasome [13], [14]. Conversely, iron starvation triggers a switch of IRP1 from cytosolic aconitase to an IRE-binding protein by loss of its [4Fe-4S] cluster, and promotes stabilization of IRP2 following degradation of FBXL5. The iron-dependent [4Fe-4S] cluster switch of IRP1 readily occurs under standard cell culture conditions with 21% oxygen, but appears less efficient al lower (3%–6%) oxygen levels that likely reflect physiological tissue oxygenation [15]. In line with this notion, IRP1 is predominantly expressed as aconitase in animal tissues, and only a small fraction is activated for IRE-binding in response to iron deficiency [16]. These findings support the idea that in vivo, the expression of major IRE-containing mRNAs, such as those encoding TfR1 and ferritin, is primarily regulated by IRP2. IRP1 may serve as a potential reservoir for further IRE-binding activity, but possibly also exert more specialized functions for the control of other IRE-containing transcripts [17].

Considering that iron is essential for cell proliferation [18], we previously hypothesized that manipulations in the IRE/IRP system to misregulate iron homeostasis may affect tumor growth. To address this, we utilized human H1299 lung cancer cells engineered for tetracycline-inducible expression of an IRP1 transgene, and assessed their capacity to form tumor xenografts in nude mice. We reported that the overexpression of IRP1 (either wild type, or a mutant carrying a C437S substitution that has constitutive IRE-binding activity) drastically inhibits the growth of tumor xenografts, without grossly affecting their iron content [19].

Here, we examine the effects of IRP2 in this model. We show that overexpression of IRP2 elicits an opposite phenotype and profoundly stimulates tumor growth, even though both IRPs appear to regulate IRE-containing mRNAs within the tumors in a similar manner. In addition, we provide evidence that the tumor-promoting activity of IRP2 requires its specific insert of 73 amino acids. Finally, we identify distinct gene expression patterns between control tumors and those overexpressing IRP2 or IRP1, that may account for their differential growth phenotypes.

## Results

### Overexpression of IRP2 promotes the growth of tumor xenografts in nude mice

**Figure 1:**
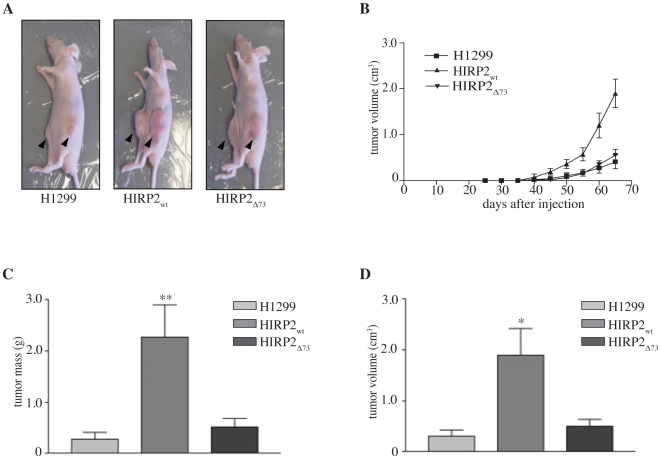
IRP2-dependent accelerated tumor growth, mediated by its specific 73 amino acids insert. BALB/c nude mice were injected with parent H1299, HIRP2_wt_ or HIRP2_Δ73_ cells and tumor xenografts were grown for 10 weeks and monitored over time. (A) Representative anesthetized mice before sacrifice; tumor xenografts are shown by arrows. (B–D) Cumulative data from three independent experiments (n = 9 mice for each group) depicting kinetics on tumor xenograft growth (B), mass (C) and volume (D) of isolated tumor xenografts. Data are expressed as mean ± SEM. * p<0.05, ** p<0.01 versus H1299 (Student's t-test).

To assess the role of IRP2 in tumorigenesis, we employed H1299 lung cancer cells overexpressing the wild type form of the protein (HIRP2_wt_) under the control of a tetracycline-inducible promoter. IRP2-transfectants and control parent cells were injected subcutaneously into the flanks of BALB/c (nu/nu) mice to form solid tumor xenografts (Fig. 1A). Palpable tumors were detectable within 3–4 weeks post injection, and their volume was monitored over time during the exponential growth phase. We noted that tumors derived from IRP2-overexpressing cells were growing much faster compared to controls (Fig. 1B). All mice were sacrificed 10 weeks post injection and the tumors were excised for biochemical and histological analysis. Tumors derived from IRP2-overexpressing cells were profoundly larger, with 5.4-fold higher average mass (Fig. 1C) and 4.6-fold higher average volume (Fig. 1D) compared to controls. These data are consistent with an apparent pro-oncogenic activity of IRP2.

**Figure 2:**
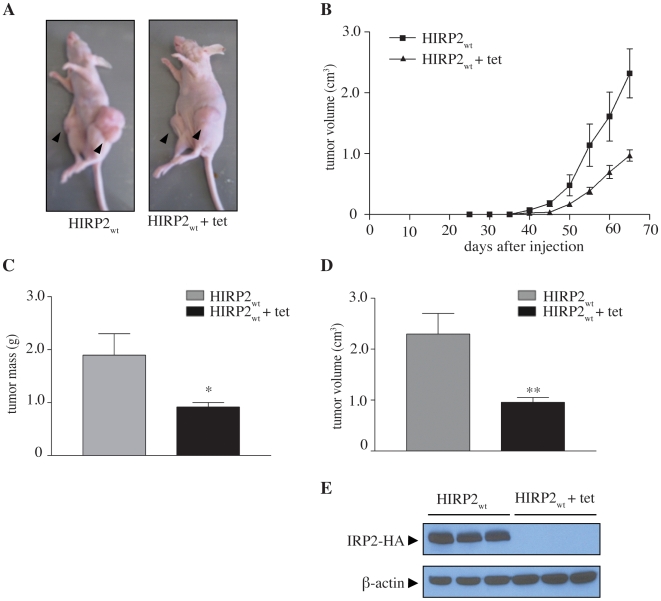
Tetracycline-dependent repression of the IRP2 transgene abolishes accelerated tumor growth. A total of 6 BALB/c nude mice were injected with HIRP2_wt_ cells to form tumor xenografts. Half of the animals were receiving 2 mg/ml tetracycline in the drinking water throughout the experimental period, starting 4 days before injection. (A) Representative anesthetized mice from the two groups before sacrifice (tumors shown by arrows). (B) Kinetics on tumor xenograft growth. (C) Mass and (D) volume of isolated tumor xenografts. (E) Tumor tissue extracts were analyzed by Western blotting with antibodies against HA, to detect expression of the IRP2 transgene, and β-actin, as loading control. Data are expressed as mean ± SEM. * p<0.05, ** p<0.01 versus HIRP2_wt_ (Student's t-test).

To exclude the possibility that the observed phenotype is due to a possible clonal effect unrelated to IRP2, further BALB/c (nu/nu) mice were injected with HIRP2_wt_ cells. Half of the animals were receiving tetracycline in their drinking water throughout the experimental period, to turn off the expression of the IRP2 transgene, while the other half were allowed to overexpress IRP2 in the xenograft, without antibiotic (Fig. 2A). The rate of tumor growth was substantially reduced in tetracycline-treated mice (Fig. 2B). Moreover, the average mass and size of tumors from these animals were 2.1- and 2.4-fold smaller, respectively, as compared to untreated counterparts (Figs. 2C–D). The expression of HA-tagged IRP2 was undetectable by Western blot analysis of tumor extracts with a HA antibody (Fig. 2E), confirming the efficient turnoff of the tetracycline promoter with the antibiotic. The reversion of the tumor growth phenotype by tetracycline validates the pro-oncogenic function of IRP2 in this xenograft model.

### The tumor-promoting activity of IRP2 requires its specific 73 amino acids insert

Mammalian IRP2 molecules contain a unique conserved insert of 73 amino acids close to their N-termini, that is absent in IRP1. The function of this sequence remains largely unknown. To better understand the requirements for the apparent pro-oncogenic activity of IRP2, we evaluated the performance of HIRP2_Δ73_ cells, overexpressing the IRP2_Δ73_ deletion mutant, in the tumorigenicity assay. These cells formed solid tumor xenografts in nude mice, that exhibited growth characteristics of control tumors derived from parent H1299 cells. Thus, in contrast to wild type protein, IRP2_Δ73_ failed to drastically stimulate tumor growth and increase tumor mass and size (Fig. 1), suggesting that the 73 amino acids insert of IRP2 is necessary for its apparent pro-oncogenic function.

Interestingly, this insert also accounts for differential anchorage-independent growth characteristics between HIRP2_wt_ and HIRP2_Δ73_ cells in soft agar. Thus, the former give rise to fewer but larger colonies compared to parent cells, while the latter form a big number of smaller size colonies (Fig. S1). To address whether the 73 amino acids insert of IRP2 suffices to elicit pro-oncogenic responses in vivo, we employed HIRP2_ΔD4_ and HIRP2_ΔD4/−73d_ cells for the tumorigenicity assay in nude mice. These cells express an inactive truncated version of IRP2, lacking the entire C-terminal domain 4, in the presence (IRP2_ΔD4_) or absence (IRP2_ΔD4/−73d_) of the 73 amino acids insert (Fig. S2, A–B). Both cell types formed slow-growing tumor xenografts in nude mice with an indistinguishable kinetic and macroscopic phenotype (Fig. S2, C–E). We conclude that the 73 amino acids domain of IRP2 is necessary but not sufficient to promote tumor xenograft growth in nude mice.

### Tumor histology

**Figure 3:**
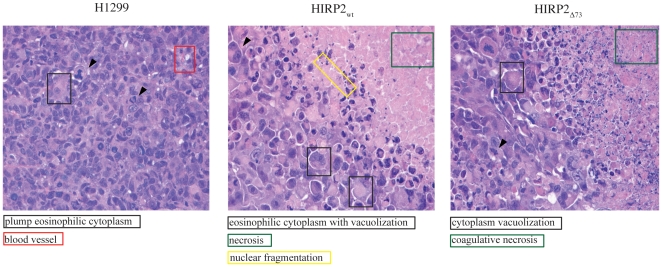
Hematoxylin and eosin staining of tumor xenografts derived from H1299 (left), HIRP2_wt_ (middle) or HIRP2_Δ73_ (right) cells (40× magnification). Mitoses are shown by arrows; colored insets indicate eosinophilic cytoplasm, blood vessels, necrosis, nuclear fragmentation, or cytoplasm vacuolization.

Histological sections of tumor xenografts were stained with hematoxylin and eosin (Fig. 3). Control tumors (derived from parent H1299 cells) contain a solid sheet of cells (Fig. 3, left), throughout which vascular channels are visible. They appear to have well-defined cytoplasmic boundaries, a variable amount of eosinophilic cytoplasm and nuclei that are pleomorphic, with about 2-fold anisokaryosis. Mitoses are in excess of 12 high-power field. Tumors derived from HIRP2_wt_ cells (Fig. 3, middle) are characterized by ∼50% of necrotic mass with mostly coagulative necrosis, that forms irregularly shaped serpentine regions. At the border between viable and necrotic tissue there is pyknosis and nuclear fragmentation. The viable cells form a diffuse sheet without architectural sophistication or differentiation and there is no intervening stroma. No blood vessels are seen within this mass. The cells have an abundance of eosinophilic cytoplasm that is often finely vacuolated. Nuclei are very large with about 10-fold anisokaryosis and mitoses are 3 per high-power field. Tumors derived from HIRP2_Δ73_ cells (Fig. 3, right) exhibit ∼20% of necrotic mass with coagulative necrosis and nuclear fragmentation at the border between viable and necrotic tissue. There are some blood vessels present in this mass and only near the periphery. Perl's iron staining was negative for all xenografts.

### Biochemical analysis of tumor xenografts and cell lines

**Figure 4:**
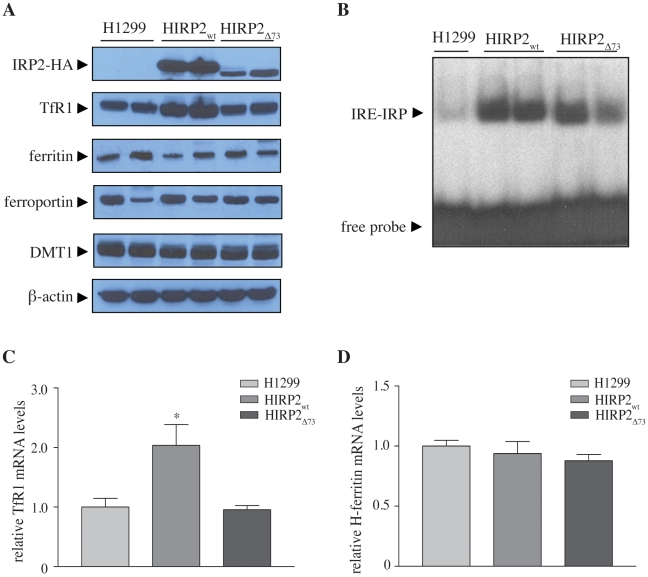
Effects of wild type or mutant IRP2 transgenes in the expression of known downstream targets within the tumor xenograft. (A) Extracts from tumor tissue were analyzed by Western blotting with antibodies against HA, TfR1, ferritin, ferroportin, DMT1 and β-actin. (B) Tumor extracts were analyzed for IRE-binding activity by EMSA with a ^32^P-labeled ferritin IRE probe. (C and D) Analysis of TfR1 and H-ferritin mRNA expression by qPCR.

Analysis of tumor extracts by Western blotting with a HA antibody (Fig. 4A, top panel) demonstrates the continuous expression of wild type or mutant IRP2 in the tumors derived from HIRP2_wt_ or HIRP2_Δ73_ cells, respectively. We noticed that IRP2_Δ73_ was expressed at lower levels than IRP2_wt_ (compare lanes 3–4 with 5–6). To examine whether this could account for the striking differences in the tumor growth phenotype (Fig. 1), we normalized the tumor volumes to relative expression levels of wild type or mutant protein from all available experimental data. The graph in Fig. S3A demonstrates that the lack of pro-oncogenic activity of IRP2_Δ73_ is genuine and unrelated to its relatively lower expression.

Tumor extracts containing either IRP2_wt_ or IRP2_Δ73_ exhibited high IRE-binding activity in an electrophoretic mobility shift assay (EMSA) (Fig. 4B). Nonetheless, the expression of only wild type but not mutant protein correlated with a significant 2-fold increase in TfR1 mRNA levels (Fig. 4C), accompanied by ∼1.6-fold higher TfR1 protein content (Fig. 4A, second panel). Interestingly, neither IRP2_wt_ nor IRP2_Δ73_ affected the expression of ferritin, ferroportin or DMT1 (Fig. 4A), by analogy to earlier observations in IRP1 xenografts [19]. A cumulative quantification of Western blots from all available experiments is depicted in Fig. S3B. As expected, ferritin mRNA levels were similar in all tumors (Fig. 4D).

**Figure 5:**
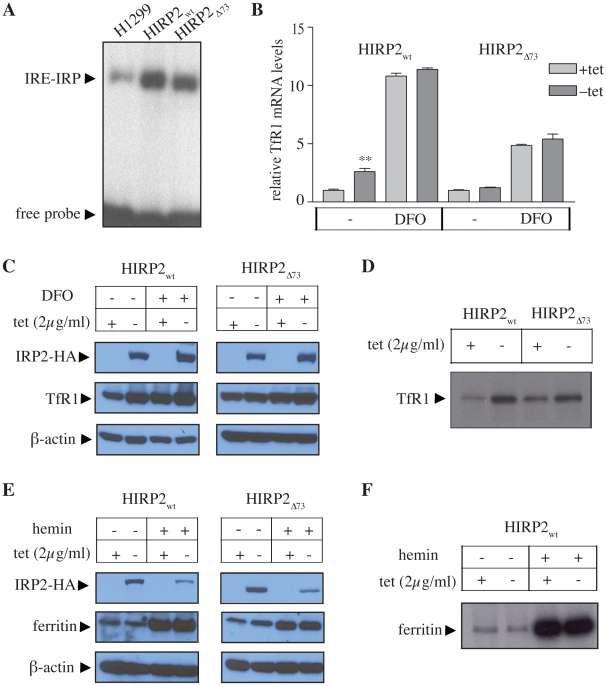
Effects of wild type or mutant IRP2 transgenes in the expression of known downstream targets in cultured H1299 cells. Parent H1299, HIRP2_wt_ and HIRP2_Δ73_ cells were grown for 3 days in the absence or presence of tetracycline; where indicated, the cells were treated overnight with 100 µM of desferrioxamine (DFO) or hemin. (A) Cytoplasmatic extracts were analyzed by EMSA with a ^32^P-labeled ferritin IRE-probe. (B) Analysis of TfR1 mRNA expression by qPCR. (C) Western blotting with antibodies against HA, TfR1 and β-actin. (D) The cells were metabolically labeled with ^35^S-methionine/cysteine and the synthesis of TfR1 was assessed by quantitative immunoprecipitation. (E) Western blotting with antibodies against HA, ferritin and β-actin. (F) The cells were untreated or pretreated for 4 h with 100 µM hemin and, subsequently, metabolically labeled with ^35^S-methionine/cysteine; the synthesis of ferritin was assessed by quantitative immunoprecipitation.

We also performed a biochemical analysis of cultured HIRP2_wt_ and HIRP2_Δ73_ cells, to explore whether the above findings in the xenograft tissue are due to basic IRP2 overexpression at the cellular level, or possibly reflect more complex responses within the tumor microenvironment. Extracts of both HIRP2_wt_ and HIRP2_Δ73_ cells were highly active in IRE-binding (Fig. 5A), as observed earlier [20]. By analogy to the data in Fig. 4, the overexpression of wild type but not mutant IRP2 in cells correlated with increased TfR1 mRNA (Fig. 5B) and protein levels (Fig. 5C), as well as TfR1 synthesis (Fig. 5D). The apparent IRP2_wt_-mediated protection of TfR1 mRNA did not suffice to increase its steady state levels to the same extent as a treatment with the iron chelator desferrioxamine (DFO), which is also known to stimulate TfR1 mRNA transcription [21]; similar results were obtained with H1299 cells expressing IRP1_C437S_ [21]. Surprisingly, neither wild type, nor mutant IRP2 suppressed ferritin expression (Fig. 5E) and synthesis (Fig. 5F) despite their capacity to bind to ferritin IRE, at least in vitro (Fig. 5A). We conclude that the biochemical data in the xenografts recapitulate IRE/IRP responses in the HIRP2_wt_ and HIRP2_Δ73_ cells.

**Figure 6:**
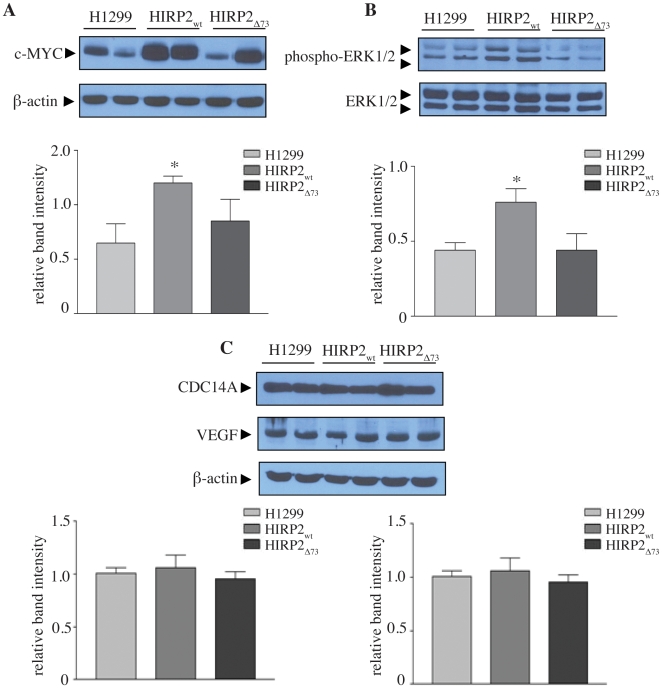
Tumor xenografts derived from HIRP2_wt_ cells display an increase of c-MYC expression and ERK1/2 phosphorylation. Extracts from tumor tissue were analyzed by Western blotting with antibodies against c-MYC, phospho-ERK1/2, ERK1/2, CDC14A, VEGF and β-actin. Representative immunoblots and quantification from three independent experiments (n = 9 mice)of (A) c-MYC, relative to β-actin; (B) phospho-ERK1/2, relative to ERK 1/2; (C) CDC14A and VEGF, relative to β-actin. Data are expressed as means of relative band intensity ± SEM. * p<0.05 versus H1299 (Student's t-test).

Further biochemical analysis of the xenografts revealed a consistent and statistically significant 2-fold increase of c-MYC expression and ERK1/2 phosphorylation in tumors derived from HIRP2_wt_ cells (Figs. 6A and B). By contrast, IRP2_Δ73_-containing tumors exhibited considerable variability in c-MYC levels and no difference in ERK1/2 phosphorylation. Neither wild type nor mutant IRP2 affected the expression of cell division cycle 14A protein (CDC14A) or vascular endothelial growth factor (VEGF) (Fig. 6C). The CDC14A transcript is a potential target of IRPs [22] and VEGF is transcriptionally induced by hypoxia inducible factors (HIF), which are also associated with the IRE/IRP regulatory system [17], [23].

### Analysis of gene expression profiles in tumor xenografts

**Figure 7:**
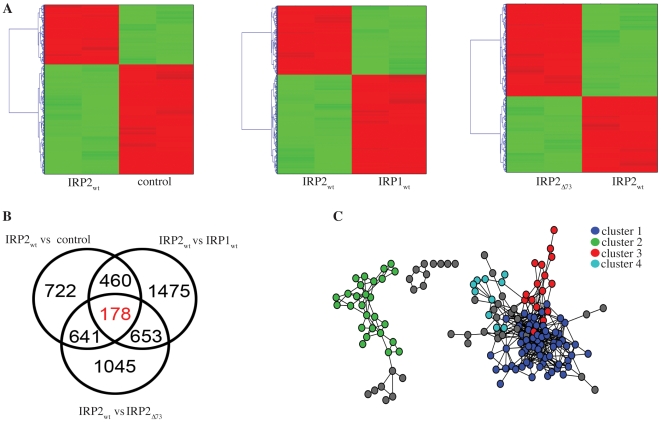
Analysis of gene expression profiles in tumor xenografts with altered expression of IRPs. (A) Hierarchical clustering of all differentially expressed genes. Red and green colors represent up- and down-regulation. (B) Venn diagram of all differentially expressed genes. (C) Co-expression network of 178 genes shared by all differential groups. Each gene is represented by a node and a Pearson correlation coefficient above 0.90 between any pair of genes is represented by an edge. Genes are colorized according to their distinct MCL cluster which may reflect a shared biological function.

Duplicate RNA samples (each from different animal) isolated from control, IRP2, IRP2_Δ73_ or IRP1 [19] tumors xenografts were subjected to cDNA microarray analysis. An array quality control assessment of sample reproducibility by pairwise PCC calculations excluded the presence of outliers among experimental replicates. The normalized dataset was further filtered by removing transcripts with low intensity values and low across-sample variances; thus, 29892 transcripts were reduced to 26903. Student t-test was applied to identify differentially expressed genes between “IRP2 vs control”, “IRP2 vs IRP1”, and “IRP2 vs IRP2_Δ73_” tumor xenografts (Fig. 7A). Genes with p-value ≤0.05 were considered significant. As expected, the expression of the TFRC gene (encoding TfR1) was increased in IRP2- as compared to control and IRP2_Δ73_-tumors (Fig. S4). “IRP2 vs control” had 2001 differentially expressed genes, “IRP2 vs IRP1”: 2766, and “IRP2 vs IRP2_Δ73_”: 2517 (Fig. 7B; a full list is provided in Table S2). There were 178 genes common to all differentially expressed groups. Differential expression was validated by qPCR in randomly selected genes (listed on Table S1).

To identify putative functional relationships, all-against-all PCCs were calculated for all common 178 genes across the dataset. Pairwise gene co-expressions were used to construct an undirected unweighted network such that genes are represented by nodes and co-expression with PCC≥0.90 by edges. The final network consisted of 156 genes (Table S3) and 546 co-expressions. 22 genes were not strongly correlated and were excluded from the network. MCL algorithm, an efficient, unsupervised, and accurate graph clustering approach based on graph flow simulation [24], was applied to group genes that may share a common biological function. This approach identified 4 clusters with more than 10 genes (Fig. 7C). The largest cluster contained 63 genes (Table S3).

Further analysis of each MCL cluster for overrepresented GO-BP terms uncovers that IRP1 and IRP2 elicit substantially distinct downstream responses. Moreover, the overexpression of IRP2, but not of IRP2_Δ73_, promotes differential expression of a wide range of genes primarily involved in signal transduction, transcriptional regulation and G-protein coupled receptor signaling, but also in metabolic processes, cell adhesion and growth (Fig. S5). Principal component analysis demonstrates that gene expression profiles between control and IRP2_Δ73_-expressing tumors exhibit remarkable similarities (Fig. S6), consistently with their growth and biochemical phenotypes.

## Discussion

Prompted by the inhibitory effects of IRP1 in tumor xenograft growth in nude mice [19], we examined the role of IRP2 in this setting. The data in Fig. 1 show that IRP2 triggers opposite responses and profoundly promotes tumor growth. Importantly, a causal relationship is established by the reversion of this phenotype with tetracycline, that efficiently turns off the expression of the IRP2 transgene in vivo (Fig. 2). Even though the administration of tetracycline clearly inhibited the pro-oncogenic function of IRP2, the respective tumors were not growing as slow as controls (compare Figs. 1 and 2). These small differences may be related to experimental variability and/or minimal leakiness of the tetracycline promoter (undetectable by immunoblotting).

In contrast to the wild type protein, the deletion mutant IRP2_Δ73_ that lacks an IRP2-specific insert of 73 amino acids, failed to stimulate tumor growth in the xenograft model (Fig. 1). Analysis of the ratios of tumor volumes to the expression of HA-tagged IRP2_wt_ and IRP2_Δ73_ (Fig. S3A) excludes the possibility that this is due to the relatively reduced expression of the mutant in tumors. The IRP2-specific insert appears to be unstructured [25] and, contrary to earlier assumptions, is dispensable for iron-dependent degradation of the protein [13], [14], [20], [26]. Our data provide strong evidence that the 73 amino acids insert confers an apparent pro-oncogenic activity to IRP2, and assign for the first time a functional role for this unique sequence. The differential anchorage-independent growth phenotypes of H1299 cells expressing either IRP2_Δ73_ or IRP2_wt_ (Fig. S1) also imply a functional significance of this sequence in cell proliferation. Nevertheless, the 73 amino acids insert failed to accelerate tumor xenograft growth when expressed outside the context of full-length and functional IRP2 (Fig. S2). Thus, the 73 amino acids insert is necessary but not sufficient to elicit pro-oncogenic responses in vivo.

Tumors derived from IRP2-overexpressing cells, as well as these cells in culture, maintained high levels of IRP2 at the experimental endpoint and exhibited an upregulation of TfR1 mRNA and protein (Figs. 4 and 5), in line with the function of IRP2 as stabilizer of TfR1 mRNA. Interestingly, while IRP2_Δ73_ was likewise highly expressed, it failed to upregulate TfR1. Considering that this deletion mutant exhibits IRE-binding activity in vitro with a ferritin IRE-probe (Figs. 4 and 5), we speculate that it may bind with reduced affinity to TfR1 IREs in vivo.

Surprisingly, the presence of the IRP2_wt_ (or IRP2_Δ73_) transgene did not affect the expression of ferritin neither in xenografts, nor in cultured cells (Figs. 4 and 5), even though IRPs are well-established inhibitors of ferritin mRNA translation. Since the tumor xenografts consist primarily of H1299 cells (Fig. 3), it is unlikely that a putative repressor activity of IRP2 is masked by high ferritin levels in stroma cells. In addition, no IRP2-dependent alteration in ferritin mRNA levels was observed, that could antagonize and mitigate the expected translational repression. Similar unexpected findings were previously documented in tumor xenografts overexpressing IRP1 [19], while the overexpression of IRP1 only conditionally suppressed ferritin mRNA translation in cultured H1299 cells grown at low densities [21]. We previously reported that at higher cell densities, ferritin mRNA bypassed the inhibitory activity of tetracycline-inducible IRP1 and was efficiently translated [21]. Analogous results were obtained with tetracycline-inducible IRP2, when HIRP2_wt_ cells were freshly generated (Guohua Chen and Kostas Pantopoulos, unpublished observations). However, after several years of maintenance of the cell lines in the lab, we are unable to observe any repression of ferritin synthesis by IRP2 (or IRP1) at low cell densities (Fig. 5). We suspect that the cells have adapted to the presence of exogenous IRP2 (or IRP1), even under suppressive conditions with tetracycline, presumably due to low leakiness of the tetracycline promoter.

The data in Fig. 6 indicate that within the tumor xenograft, IRP2 (or IRP2_Δ73_) may also fail to control the expression of other IRE-containing mRNAs, such as those encoding DMT1, ferroportin and CDC14A. Nevertheless, we cannot exclude the possibility that all these proteins are encoded by non-IRE-containing mRNA isoforms, generated by alternative splicing [4], [5], [22]. The absence of any IRP2-dependent alterations in VEGF levels may suggest that the H1299 cells primarily express HIF-1α, that is unresponsive to IRPs. In any case, this finding is concordant to the lack of vascularization in the necrotic tumor mass (Fig. 3).

We noted that the expression of the IRP2 transgene in tumor xenografts was associated with increased levels of the c-MYC oncogene, as well as with increased ERK1/2 phosphorylation (Figs. 6A and B). This was not the case with IRP2_Δ73_, even though a trend towards higher c-MYC content was evident in IRP2_Δ73_-related tumors, without reaching statistical significance. The expression of c-MYC is regulated at multiple levels and deregulation of this gene is associated with malignant transformation and cancer [27]. Our cDNA microarray analysis did not show any increase in c-MYC mRNA levels. Thus, we speculate that the IRP2-dependent increase in c-MYC protein content may be related, at least partially, with stabilization of this protein following phosphorylation by ERK1/2 [28].

The IRP2-dependent increase in c-MYC levels deserves particular attention, considering that IRP2 is a direct transcriptional target of c-MYC [29]. Our data are consistent with a regulatory feedback loop between c-MYC and IRP2 that controls tumor growth. It was proposed that the c-MYC-mediated transcriptional activation of IRP2, and concomitant transcriptional and translational suppression of H-ferritin by c-MYC and IRP2, respectively, contribute to cell transformation by increasing the intracellular iron pool [29]. In the tumor xenograft model presented here, the pro-oncogenic properties of IRP2 are independent of its capacity to regulate ferritin expression and tumor iron levels. In line with this notion, the tumor suppressor phenotype associated with IRP1 overexpression in tumor xenografts was also independent of ferritin and iron levels [19].

Taken together, our data provide strong evidence that the apparent pro-oncogenic activity of IRP2 is unrelated to the established function of this protein as regulator of several known IRE-containing mRNAs. The cDNA microarray analysis (Fig. 7) demonstrates that the overexpression of either IRP2 or IRP1 transgenes is associated with distinct gene expression patterns in tumors, that may account for their strikingly opposite growth phenotypes. Furthermore, our data suggest that the unique 73 amino acids insert confers to IRP2 pro-oncogenic potential and is essential for accelerated tumor growth. This view is reinforced by the microarray results, that uncover considerable similarities in gene expression profiles of IRP2_Δ73_ and control tumors. Elucidating the biochemical function of the 73 amino acids insert will be important to understand the mechanism underlying the pro-oncogenic activity of IRP2.

## Materials and Methods

### Cell culture

Human H1299 lung cancer cells and clones expressing wild type IRP2 (HIRP2_wt_), wild type IRP1 (HIRP1_wt_) or the IRP2 deletion mutants Δ73 (HIRP2_Δ73_), ΔD4 (HIRP2_ΔD4_) or ΔD4/-73d (HIRP2_ΔD4/-73d_) in a tetracycline-inducible fashion (tet-off) were grown in Dulbecco's modified Eagle medium (DMEM) containing 10% fetal bovine serum, 2 mM glutamine, 100 U/ml penicillin and 0.1 mg/ml streptomycin (Wisent Inc, St-Bruno, QC). The generation of these cell lines has been described elsewhere [20], [30], [31]. Stable clones were maintained in the presence of 2 µg/ml tetracycline, 2 µg/ml puromycin and 250 µg/ml G418. Expression of transfected proteins was induced by removal of tetracycline from media supplemented with tetracycline-free fetal bovine serum (Clontech).

### Soft agar colony formation assay

The capacity of HIRP2_wt_ and HIRP2_Δ73_ cells to form colonies in soft agar was evaluated as earlier described [19].

### Animal experiments

All experimental procedures were approved by the Animal Care Committee of McGill University (Protocol 4966). Female BALB/c (nu/nu) mice were obtained from Charles River Laboratories (Cambridge, MA). The animals were housed in macrolone cages (up to 5 animals/cage, 12:12 h light-dark cycle: 7 am – 7 pm; 22±1°C, 60±5% humidity) according to standard guidelines, and had free access to water and food. Tumor xenografts were formed as described in [19]. The mice were monitored three time a week for tumor growth for up to ten weeks. All animals were euthanized before developing any behavioral signs of disease. When indicated, animals received tetracycline in the drinking water (2 mg/ml) 4 days before cell injection and throughout the duration of the experiment; the solution was refreshed every second day.

### Histological analysis

Tissue staining with hematoxylin and eosin, and Perl's Prussian blue was performed as in [19].

### Preparation of cell and tumor tissue extracts

Cells were washed twice in cold PBS and lysed in a buffer containing 20 mM Tris-Cl pH 7.4, 40 mM KCl, 1% Triton X-100, an EDTA-free protease inhibitor cocktail (Roche) and a Halt phosphatase inhibitor Cocktail (Thermo Scientific). Frozen tumor tissue aliquots were suspended in the same lysis buffer and homogenized in a 1 ml glass homogenizer. Cell debris was cleared by centrifugation and the protein concentration was measured with the Bradford reagent (BioRad).

### Western blotting

Protein extracts (25–50 µg) were resolved by SDS-PAGE on 8%, 10% or 14% gels and the proteins were transferred onto nitrocellulose filters (BioRad). The blots were saturated with 10% non-fat milk in PBS containing 0.1% (v/v) Tween-20 (PBS-T) and probed with hemagglutinin (HA) (Roche), TfR1 (Zymed), ferritin (Novus), ferroportin (raised in a rabbit against an affinity-purified GST-fusion protein antigen containing 4 tandem copies of the C-terminal 32 amino acid domain of mouse ferroportin), DMT1 [32] c-MYC or VEGF (Santa Cruz), phospho-ERK1/2 or ERK1/2 (Cell Signaling), CDC14A (R&D), or β-actin (Sigma) antibodies. A 1∶1000 dilution was used for all antibodies expect that for ferritin, which was diluted 1∶500. After three washes with PBS-T, the blots were incubated with peroxidase-coupled goat anti rat IgG (Roche) for the HA antibody, peroxidase-coupled rabbit anti-mouse IgG (Sigma) for the TfR1, c-MYC and β-actin antibodies, peroxidase-coupled goat anti-rabbit IgG (Sigma) for the ferroportin, ferritin, phospho-ERK1/2 and ERK1/2 antibodies, and peroxidase-coupled donkey anti-goat (Santa Cruz) for the VEGF antibody. All secondary antibodies were diluted 1∶5000. Peroxidase-coupled antibodies were detected by enhanced chemiluminescence with the Western Lightning ECL kit (Perkin Elmer). Immunoreactive bands were quantified by densitometric scanning.

### Electrophoretic mobility shift assay (EMSA)

Cytoplasmic lysates were analyzed for IRE-binding activity by EMSA with a ^32^P-labeled ferritin IRE probe [33].

### Metabolic labeling and immunoprecipitation

Cells were metabolically labeled with 50 µCi/ml Trans-[^35^S]-label, a mixture of 70∶30 ^35^S-methionine/cysteine (ICN). After 2 h, the cells were lysed in a buffer containing 50 mM Tris-Cl pH 7.4, 300 mM NaCl and 1% Triton X-100. Cell debris was cleared by centrifugation and cell lysates were subjected to quantitative immunoprecipitation with ferritin or TfR1 antibodies [21]. Immunoprecipitated proteins were analyzed by SDS-PAGE. Radioactive bands were visualized by autoradiography and quantified by phosphorimaging.

### Extraction of RNA

Total RNA was isolated from frozen tumor tissue using the RNeasy Midi kit (Qiagen). The quality of RNA was assessed by determining the 260/280 nm absorbance ratios and by agarose gel electrophoresis.

### DNA microarray expression profiling

Total RNA (5 µg) was reverse transcribed using the Fairplay III Microarray Labeling kit (Stratagene) and labeled with Cy3-dCTP or Cy5-dCTP. Following clean-up using Fairplay columns (Stratagene), labeled cDNA and universal reference (Stratagene) were combined and hybridized to a 4×44K two-color gene expression array (Agilent Technologies). After washes according to the manufacturer's protocol, the array was scanned on the Agilent DNA Microarray scanner at a resolution of 5 microns. All images were extracted and normalized with Feature Extracter 9.5. The microarray data are MIAME compliant and have been deposited in the ArrayExpress database (accession number E-MEXP-2524).

### Gene co-expression network inference

Pairwise similarity in gene expression vectors was expressed by the Pearson correlation coefficient (PCC). Gene pairs that correlated above a predefined PCC threshold were represented in the form of an undirected unweighted network [34], where nodes correspond to genes and links (edges) correspond to co-expression between genes. Gene pairs with PCC ≥0.90 were considered as co-expressed. To reduce the number of false positive co-expression edges and to identify putative functionally related gene clusters, the Markov Cluster Algorithm (MCL) method was applied [24]. To assess significance of enrichment, only clusters with 10 or more genes were retained. Genes identified to be present in the same cluster were analyzed for overrepresented Gene Ontology Biological Process (GO-BP) terms.

### Quantitative real-time PCR (qPCR)

Total RNA (1 µg) was reverse transcribed using the Quantitech Reverse Trancription Kit (Qiagen). Gene-specific primers (Table S1) were used to perform qPCR on an MX 3005 Real-time PCR system (Stratagene) with the Quantitech SYBR Green PCR kit (Qiagen). The specificity of each primer set was monitored by dissociation curve analysis. All experiments were normalized using ribosomal protein S18 as housekeeping gene.

### Statistical analysis

Results are presented as mean ± SEM. Comparisons were made using unpaired student's t test. A value of P<0.05 was considered statistically significant.

## Supporting Information

Figure S1 Deletion of the 73 amino acids insert of IRP2 alters growth properties in soft agar. (A) Schematic representation of wild type IRP2 and the IRP2_Δ73_ deletion mutant, depicting the 4 domains of the protein, the 73 amino acids insert within domain 1, the hinge linking domains 3 and 4, and the C-terminal HA tag. (B) Tetracycline-inducible expression of wild type IRP2 or IRP2_Δ73_. Extracts of parent H1299, HIRP2_wt_ and HIRP2_Δ73_ cells, grown for 48 h without (−) or with (+) 2 mg/ml tetracycline, were analyzed by Western blotting with antibodies against HA (top) and β-actin (bottom). (C) Anchorage-independent growth of the cells in soft agar. Representative images of colonies derived from a total of 2×10^4^ plated cells (100× magnification) are shown on top and colony formation efficiency at the bottom. Media didn't contain tetracycline to allow expression of transfected IRP2 or IRP2_Δ73_. * p<0,001 versus H1299 (Student's t-test).
(2.83 MB TIF)

Figure S2 The 73 amino acids insert of IRP2 is not sufficient to promote tumor growth. (A) Schematic representation of wild type IRP2 and the deletion mutants lacking domain 4, either in the presence (IRP2_ΔD4_) or absence of the 73 amino acids insert (IRP2_ΔD4/−73d_). (B) Growth of tumor xenografts derived from HIRP2_ΔD4_ and HIRP2_ΔD4/−73d_ cells (n = 3 nude mice per group). (C) Detection of mutant IRP2 expression in tumor extracts by Western blotting with antibodies against HA and control β-actin. (D) Mass and (E) volume of isolated tumor xenografts. Data are expressed as mean ± SEM. The graphs are in the same scale as those in Figs. 1 and 2 to allow direct comparison.
(0.70 MB TIF)

Figure S3 The low pro-oncogenic activity of IRP2_Δ73_ is not due to reduced expression levels of this mutant in tumors. The graph depicts the ratio of tumor volume values (derived from HIRP2_wt_ and HIRP2_Δ73_ cells) by the relative band intensities of HA-tagged IRP1_wt_ and IRP2_Δ73_ (obtained by densitometric analysis of Western blots). Data are from three independent experiments (n = 9 mice); ** p<0.01 versus HIRP2_wt_ (Student's t-test). (B) Quantification of TfR1, ferritin, ferroportin and DMT1 expression in tumor xenografts derived from HIRP2_wt_ and HIRP2_Δ73_ cells. Western blots from three independent experiments were quantified by densitometry. Values of protein band intensities (mean ±SEM) were normalized to β-actin; ** p<0.01 versus H1299 (Student's t-test).
(0.91 MB TIF)

Figure S4 Box plot of the normalized TFRC (TfR1) expression values in tumor xenografts derived from parent H1299, HIRP1_wt_, HIRP2_wt_ and HIRP2_Δ73_ cells, generated by the cDNA microarray analysis.
(0.45 MB TIF)

Figure S5 Functional annotations of pairwise (“IRP2 vs control”, “IRP2 vs IRP1” and “IRP2 vs IRP2_Δ73_”) differentially regulated genes in tumor xenografts derived from parent H1299, HIRP2_wt_, HIRP1_wt_ [ref. (19)] and HIRP2_Δ73_ cells.
(0.79 MB TIF)

Figure S6 Principal component analysis. Experiments were plotted by mapping values of 1st and 2nd principal components to X- and Y- axis respectively. The distance of separation between samples corresponding to “control” and “IRP2_Δ73_” tumors is insignificant, suggesting a common signal intensity pattern.
(0.88 MB TIF)

Table S1 Gene specific primers used for qPCR experiments.
(0.06 MB PDF)

Table S2 Full list of pairwise (“IRP2 vs control”, “IRP2 vs IRP1” and “IRP2 vs IRP2_Δ73_”) differentially expressed genes in tumor xenografts derived from parent H1299, HIRP2_wt_, HIRP1_wt_ [ref. (19)] and HIRP2_Δ73_ cells.
(0.69 MB XLS)

Table S3 Annotation and network statistics for common differentially expressed genes between “IRP2 vs control”, “IRP2 vs IRP1” and “IRP2 vs IRP2_Δ73_”.
(0.15 MB XLS)
