# Supplementary figures and images for: Tumorigenic Properties of Iron Regulatory Protein 2 (IRP2) Mediated by Its Specific 73-Amino Acids Insert
Source: PLoS One. 2010 Apr 13;5(4):e10163. doi: 10.1371/journal.pone.0010163 (PMC2854138; doi:10.1371/journal.pone.0010163)

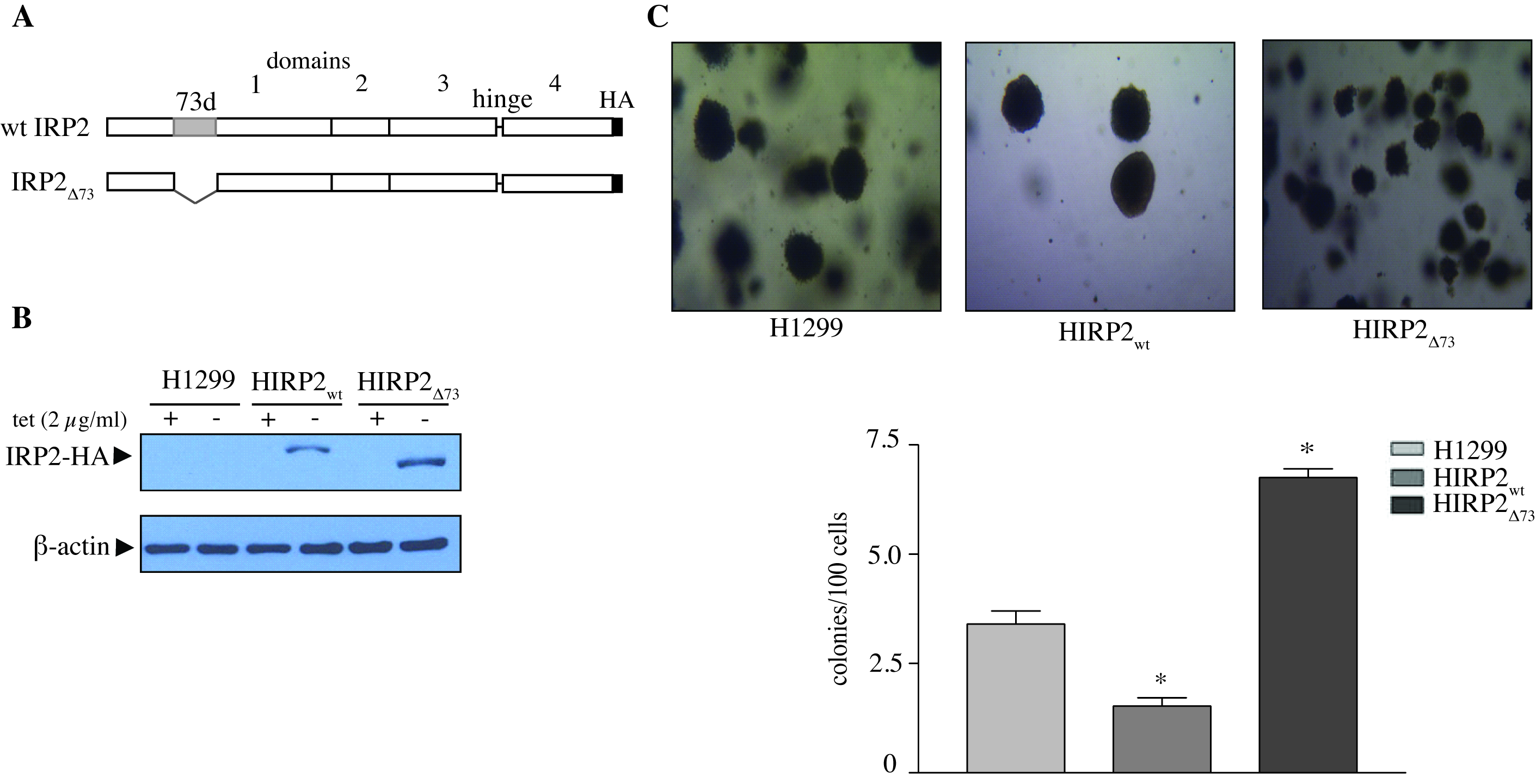

Supplement: Figure S1 — Deletion of the 73 amino acids insert of IRP2 alters growth properties in soft agar. (A) Schematic representation of wild type IRP2 and the IRP2Δ73 deletion mutant, depicting the 4 domains of the protein, the 73 amino acids insert within domain 1, the hinge linking domains 3 and 4, and the C-terminal HA tag. (B) Tetracycline-inducible expression of wild type IRP2 or IRP2Δ73. Extracts of parent H1299, HIRP2wt and HIRP2Δ73 cells, grown for 48 h without (−) or with (+) 2 mg/ml tetracycline, were analyzed by Western blotting with antibodies against HA (top) and β-actin (bottom). (C) Anchorage-independent growth of the cells in soft agar. Representative images of colonies derived from a total of 2×10^4^ plated cells (100× magnification) are shown on top and colony formation efficiency at the bottom. Media didn't contain tetracycline to allow expression of transfected IRP2 or IRP2Δ73. * p<0,001 versus H1299 (Student's t-test). (2.83 MB TIF) [file pone.0010163.s001.tif]

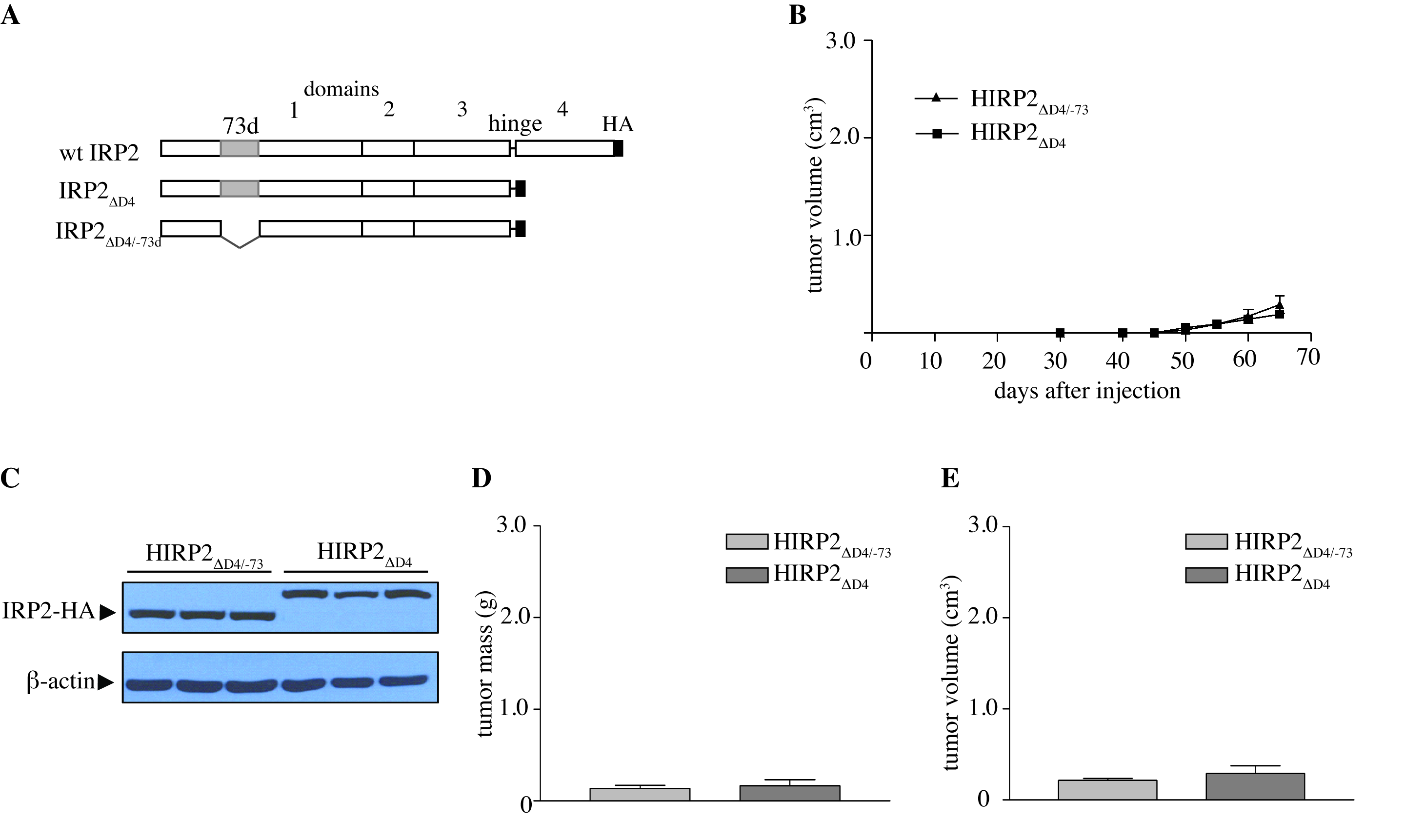

Supplement: Figure S2 — The 73 amino acids insert of IRP2 is not sufficient to promote tumor growth. (A) Schematic representation of wild type IRP2 and the deletion mutants lacking domain 4, either in the presence (IRP2ΔD4) or absence of the 73 amino acids insert (IRP2ΔD4/−73d). (B) Growth of tumor xenografts derived from HIRP2ΔD4 and HIRP2ΔD4/−73d cells (n = 3 nude mice per group). (C) Detection of mutant IRP2 expression in tumor extracts by Western blotting with antibodies against HA and control β-actin. (D) Mass and (E) volume of isolated tumor xenografts. Data are expressed as mean ± SEM. The graphs are in the same scale as those in Figs. 1 and 2 to allow direct comparison. (0.70 MB TIF) [file pone.0010163.s002.tif]

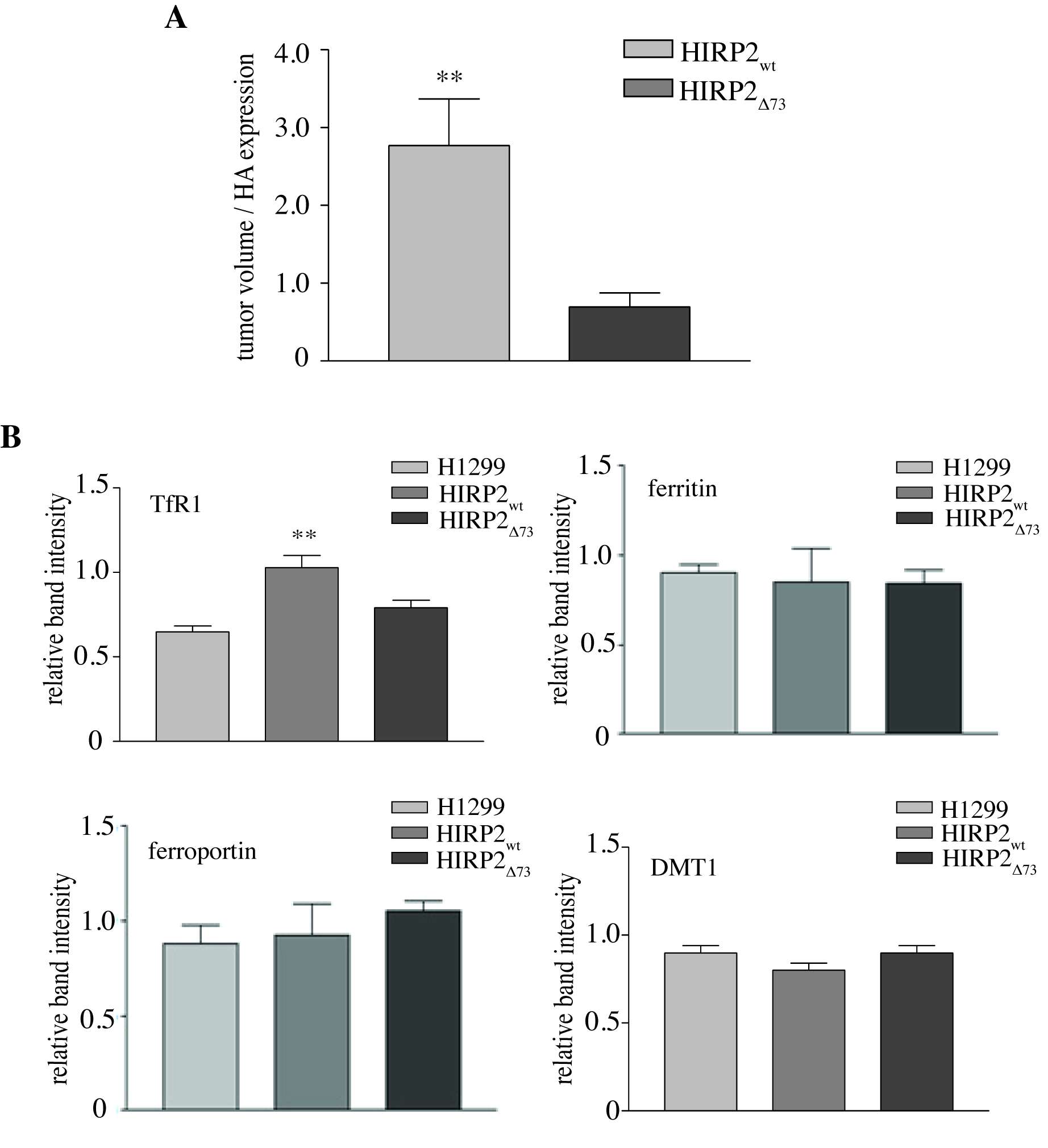

Supplement: Figure S3 — The low pro-oncogenic activity of IRP2Δ73 is not due to reduced expression levels of this mutant in tumors. The graph depicts the ratio of tumor volume values (derived from HIRP2wt and HIRP2Δ73 cells) by the relative band intensities of HA-tagged IRP1wt and IRP2Δ73 (obtained by densitometric analysis of Western blots). Data are from three independent experiments (n = 9 mice); ** p<0.01 versus HIRP2wt (Student's t-test). (B) Quantification of TfR1, ferritin, ferroportin and DMT1 expression in tumor xenografts derived from HIRP2wt and HIRP2Δ73 cells. Western blots from three independent experiments were quantified by densitometry. Values of protein band intensities (mean ±SEM) were normalized to β-actin; ** p<0.01 versus H1299 (Student's t-test). (0.91 MB TIF) [file pone.0010163.s003.tif]

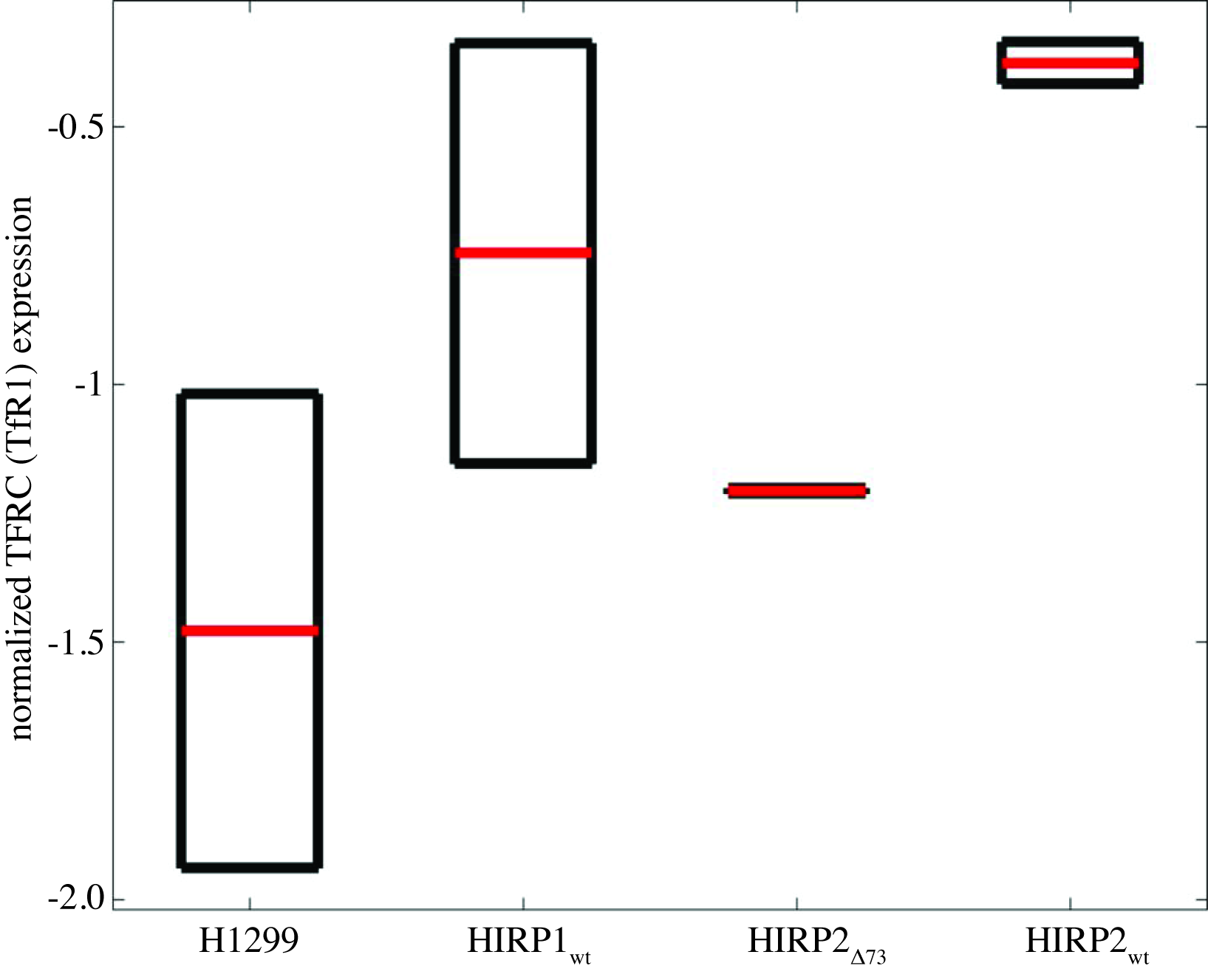

Supplement: Figure S4 — Box plot of the normalized TFRC (TfR1) expression values in tumor xenografts derived from parent H1299, HIRP1wt, HIRP2wt and HIRP2Δ73 cells, generated by the cDNA microarray analysis. (0.45 MB TIF) [file pone.0010163.s004.tif]

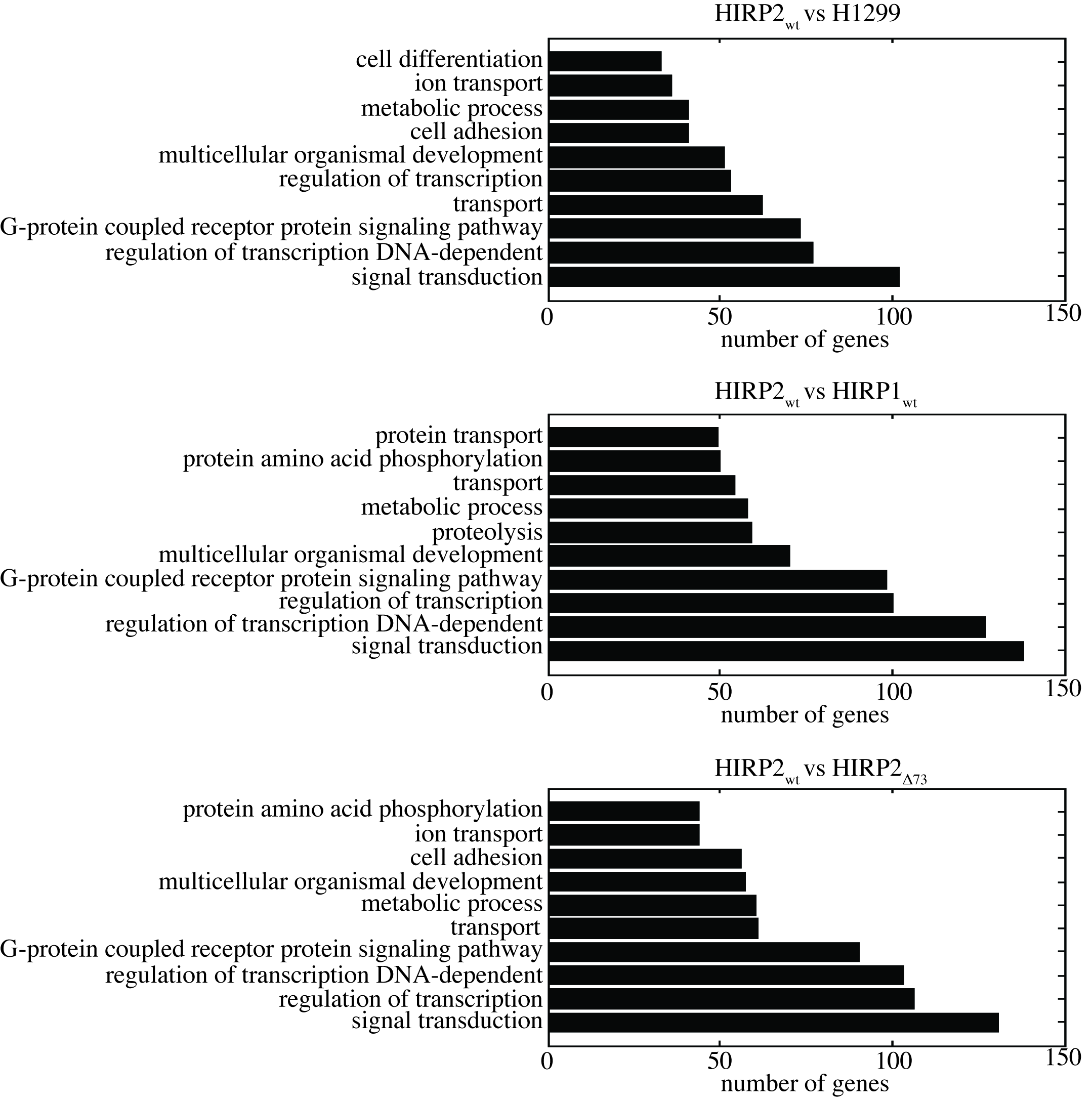

Supplement: Figure S5 — Functional annotations of pairwise (“IRP2 vs control”, “IRP2 vs IRP1” and “IRP2 vs IRP2Δ73”) differentially regulated genes in tumor xenografts derived from parent H1299, HIRP2wt, HIRP1wt [ref. (19)] and HIRP2Δ73 cells. (0.79 MB TIF) [file pone.0010163.s005.tif]

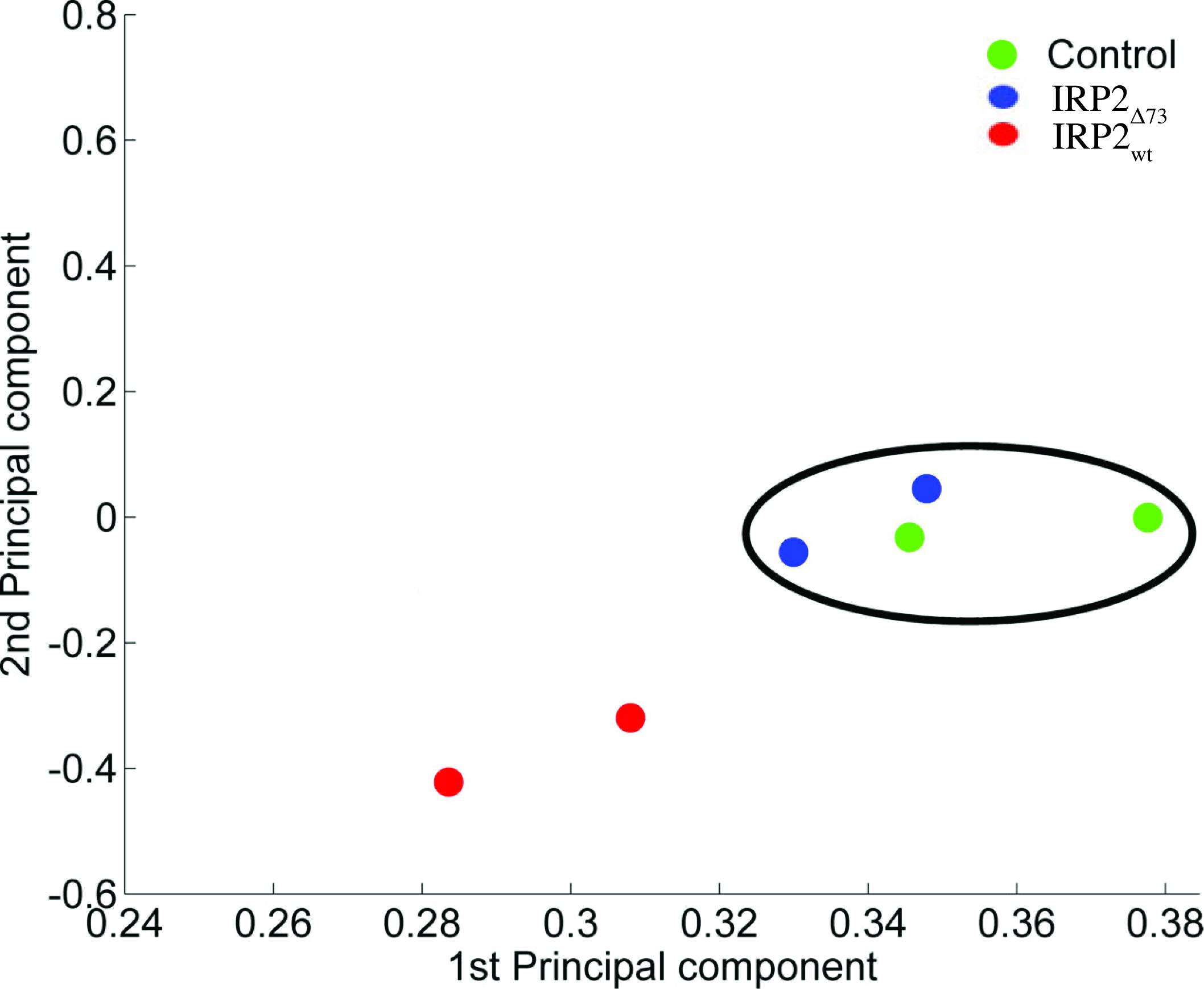

Supplement: Figure S6 — Principal component analysis. Experiments were plotted by mapping values of 1st and 2nd principal components to X- and Y- axis respectively. The distance of separation between samples corresponding to “control” and “IRP2Δ73” tumors is insignificant, suggesting a common signal intensity pattern. (0.88 MB TIF) [file pone.0010163.s006.tif]
