# Supplementary material for: Tumorigenic Properties of Iron Regulatory Protein 2 (IRP2) Mediated by Its Specific 73-Amino Acids Insert
Source: PLoS One. 2010 Apr 13;5(4):e10163. doi: 10.1371/journal.pone.0010163 (PMC2854138; doi:10.1371/journal.pone.0010163)
Supplement: Table S1 — Gene specific primers used for qPCR experiments. (0.06 MB PDF) [file pone.0010163.s007.pdf]

Table S1. Primers used for real-time PCR

**Gene name**	**Primer sequence (5 → 3')**	**Genebank accession number**	**Expected product size (bp)**
TfR1	F: GCAAGTAGATGGCGATAACAG<br>R: GACGATCACAGCAATAGTCCC	NM 003234	145
H- Ferritin	F: GAGACCACAAGCGACCCGCA<br>R: GAGGTGACGGAGGGCTGGCT	NM 002032.2	138
PTNP11	F: CCCACATCAAGATTCAGAACACT<br>R: GCCCGTGATGTTCCATGTAA	NM 002834	105
DNAJA1	F: GCTGCAACGGAAGGAAGAT<br>R: CCAGTCCTGGTTCTTGGTCT	NM 001539	120
HSPE1	F: GGTTGAAAGGAGTGCTGCTGAA<br>R: GAATGGGCAGCATCATGTTGAT	NM 002157	303
PSMC6	F: GCTGCGTCCAGGAAGATTAG<br>R: TGCGAACATACCTGCTTCAG	NM 002806	196
VCP	F: TTCCTGAAGTTTGGCATGACAC<br>R: GCGGGCCTTGTCAAAGAT	NM 007126	195
VDAC3	F: AATTTCGCCCTGGGTTACAA<br>R: TCAGTGCCATCGTTCACATGT	NM 001135694	65
RPS18	F: TGTGGTGTTGAGGAAAGCAG<br>R: AAGTGACGCAGCCCTCTATG	NM 022551	252

TfR1: transferrin receptor 1; PTNP11: tyrosine phosphatase, non-receptor type 11; DNAJA1: DnaJ (Hsp40) homolog, subfamily A, member 1; HSPE1: heat shock 10kDa protein 1 (chaperonin 10); PSMC6: proteasome 26S subunit, ATPase, 6; VCP: valosin-containing protein; VDAC3: voltage-dependent anion channel 3; RPS18: ribosomal protein S18.
